# The Cyst of the Canal of Nuck: Anatomy, Diagnostic and Treatment of a Very Rare Diagnosis—A Case Report of an Adult Woman and Narrative Review of the Literature

**DOI:** 10.3390/medicina58101353

**Published:** 2022-09-27

**Authors:** Michael Kohlhauser, Julian Vinzent Pirsch, Thorsten Maier, Christian Viertler, Roland Fegerl

**Affiliations:** 1 Department of Surgery, State Hospital Weiz, Styrian Hospital Association (KAGes), 8160 Weiz, Austria; 2 Radiological Center Weiz, Institute for CT and MRI Weiz OG, 8160 Weiz, Austria; 3 Diagnostic and Research Institute of Pathology, Medical University of Graz, 8010 Graz, Austria

**Keywords:** Nuck cyst, cyst of the canal of Nuck, hydrocele, hernia, canal of Nuck, rare diseases

## Abstract

The cyst of the canal of Nuck is an extremely rare female hydrocele, usually occurring in children, but also in adult women. It is caused by pathology of the canal of Nuck, which is the female equivalent to the male processus vaginalis. Due to its rarity and the lack of awareness among physicians, the cyst of the canal of Nuck is a seldom-encountered entity in clinical practice and is commonly misdiagnosed. We report on a case of cyst of the canal of Nuck in a 42-year-old woman, who presented with a painful swelling at her right groin. In addition, we conducted a review of the current available literature. This review gives an overview of the anatomy, pathology, diagnostics, and treatment of the cyst of the canal of Nuck. The aim of this review is not only to give a survey, but also to raise awareness of the cyst of the canal of Nuck and serve as a reference for medical professionals.

## 1. Introduction

A female hydrocele, namely cyst of the canal of Nuck, is an extremely rare entity that is not commonly encountered, especially in adults [1,2]. The origin of this disease is a pathology during embryogenesis [3,4]. Clinically, a female hydrocele typically manifests as a swelling in the groin or genital region [5,6,7,8,9,10,11,12,13,14,15,16,17,18,19,20,21,22], which allows for a variety of differential diagnoses. Due to its rarity, most health professionals are not aware of its existence and the cyst of the canal of Nuck is often misdiagnosed [7,8,9,12,23]. Precise diagnosis, including a thorough clinical examination and adequate radiological imaging, is required to accurately determine its presence. We report a case of a 42-year-old woman with a cyst of the canal of Nuck who presented to our department. This case provides insight into the various diagnostic procedures used, describes the surgical approach, and gives information about the histology. In addition, we conduct a review of the available literature to summarize the experiences of various physicians worldwide. This review provides basic knowledge about the anatomy and pathogenesis. Secondly, we provide an overview of the prevalence of the cyst of the canal of Nuck. We present commonly used classifications and describe the symptoms and key aspects of the diagnostics, which enable the distinction of possible differential diagnosis of inguinal or genital swelling in females. Furthermore, we examine the therapy and discuss different surgical approaches. This review should serve as compendium to facilitate the identification of female hydroceles and their treatment.

## 2. Methodology

Due to the extremely rare occurrence of the condition, we conducted a literature review to understand the anatomical background, the diagnostics and treatment methods in order to highlight best practice in medical care of this phenomenon. The following databases were used to search for and identify the included literature: PubMed, Google Scholar, and MEDLINE. No date restriction was imposed on the search. The result of our review is presented in narrative form in addition to our case report.

## 3. Case Report

A 42-year-old female patient was referred to the surgical outpatient department by her general practitioner due to a small swelling on the right groin, with a suspected inguinal hernia. The patient had observed this swelling two months before, and was suffering dragging pain in the affected area. With the exception of a hysterectomy and a bilateral salpingectomy by reason of multiple myoma uteri two years ago, there was neither a history of abdominal or pelvic surgery nor trauma, and the patient was otherwise in good health, without the appearance of nausea or vomiting. On physical examination, a non-reducible deep-seated round tumor was detected and located in the right inguinal region with a size of approximately 15 × 10 mm, with no pressure pain. The remaining abdominal examination was inconspicuous, with soft abdominal wall conditions and regular peristaltic sounds. There was no complaint of lower extremity weakness.

**Figure 1:**
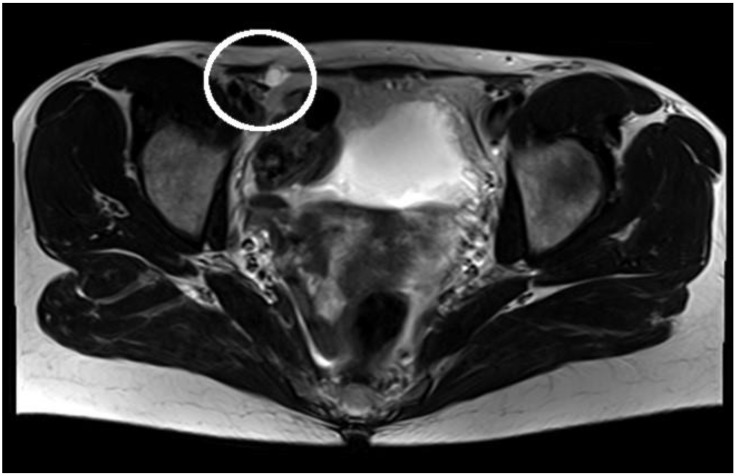
Axial T2-weighted MRI: cystic structure localized in the right inguinal canal, with no communication to the peritoneal cavity.

An ultrasound examination showed a hypoechoic cystic formation originated from the round ligament of the uterus in the right inguinal region without vascular flow or peristalsis. No changes in size or shape due to Valsalva maneuver could be determined. Magnetic resonance imaging (MRI) was obtained to further define the extent and nature of the cyst. The MRI revealed a 15 × 9 × 16 mm (transversal × sagittal × craniocaudal) well-defined, cystic formation with a peripheral contrast-enhancement within the medial border of her right inguinal ligament with no visible communication to the peritoneal cavity, compatible with a hydrocele of the Nuck canal. An axial T2-weighted MRI image of this hydrocele is displayed in Figure 1.

**Figure 2:**
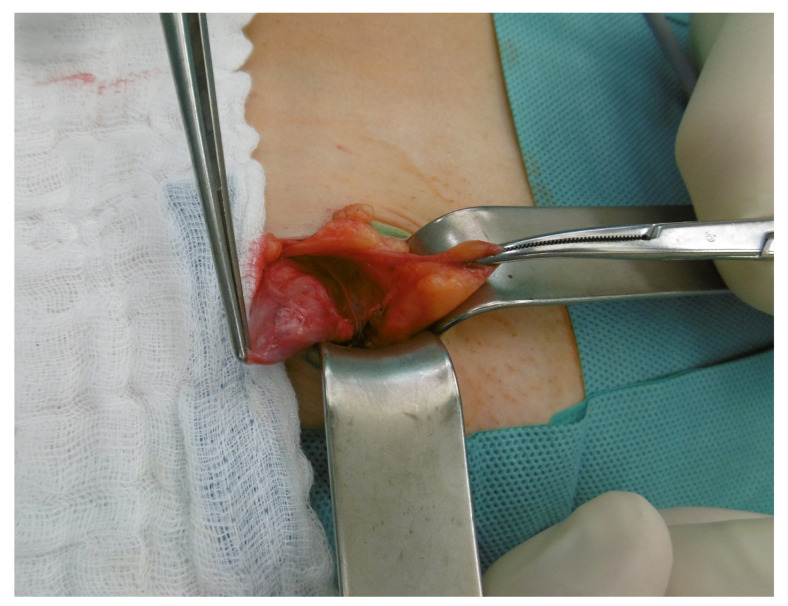
Intraoperative picture of the cyst of the canal of Nuck after dissection from the round ligament.

**Figure 3:**
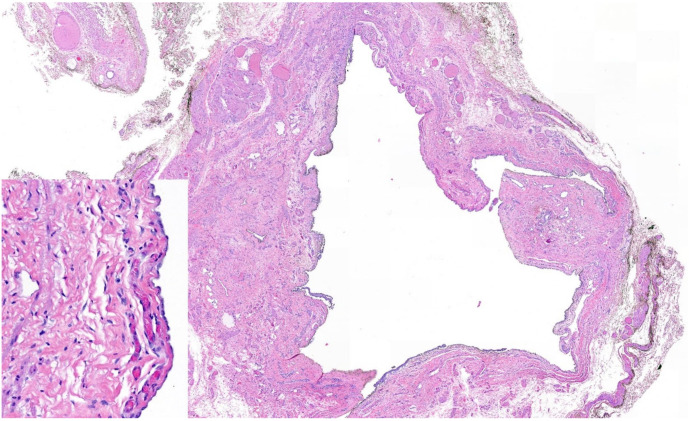
Histology hematoxylin and eosin staining: cystic structure localized within fibromuscular and adipose tissue demonstrating a flat to cuboidal mesothelial lining (left corner), consistent with a hydrocele of the canal of Nuck.

The patient was admitted to the ward for preparation for open surgery. Due to the anatomical conditions, an open surgical procedure was determined. Pre-surgically, the cyst was detected by ultrasonography, followed by a para-inguinal incision over the verified cyst formation. Intrasurgically, a small cyst appeared, between the external abdominal oblique and the right inguinal ligament. The cyst was dissected from the round ligament (Figure 2), followed by a ligation and completely excision. The internal inguinal ring defect was repaired without the use of a mesh, by suturing the tendon of the external abdominal oblique to the shelving edge of the inguinal ligament. Post-surgery, the cyst was opened with a scalpel, whereby a mucous, light-colored fluid was discharged. The histological intervention confirmed the diagnosis of cyst of the canal of Nuck. The image of the hematoxylin and eosin staining as well as a brief explanation is shown in Figure 3. In our follow-up 6 months after surgery, the patient was asymptomatic and satisfied with the treatment.

## 4. Review of Literature

### 4.1. Anatomical and Pathological Background

**Figure 4:**
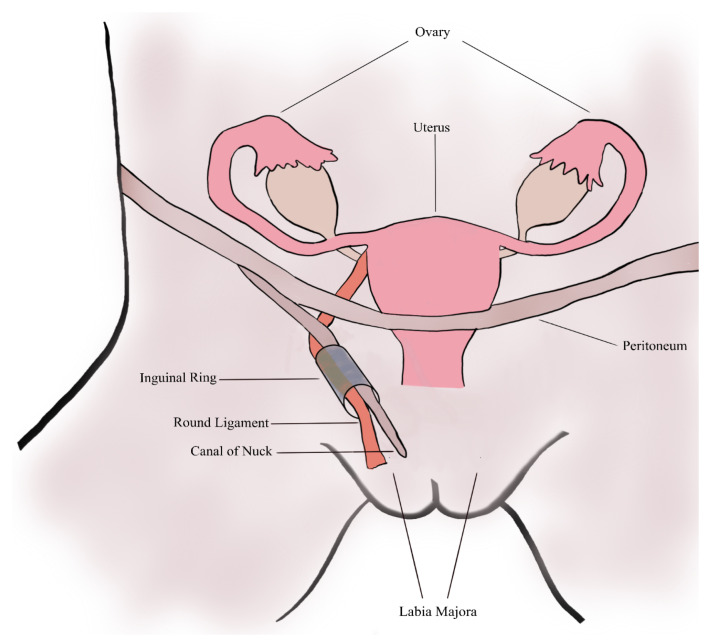
A schematic of the female anatomy and the patent canal of Nuck.

In 1691, Anton Nuck, a Dutch anatomist, was the first to describe the canal of Nuck [24]. The canal of Nuck is the female equivalent to the processus vaginalis in males, which usually disappears within the first year of life. It consists of an evagination of peritoneum, which is attached to the uterus by the round ligament, and proceeds through the inguinal ring alongside the round ligament into the labia majora [3,11]. Usually, the superior part of this outpouch obturates during or just before birth and disappears within the first year of life. In rare cases, this obturation fails, resulting in a persistence of the canal of Nuck [3,4,24] (Figure 4), which can cause the formation of a female hydrocele, namely the cyst of the canal of Nuck [1,13]. This phenomenon in women was first reported by Coley in 1892 [25]. Similar to the male hydrocele, a female hydrocele probably arises due to an imbalance of secretion from and absorption of fluid by the secretory membranes of the canal of Nuck [3,26]. Although this imbalance is most frequently idiopathic, disturbed lymphatic drainage caused by trauma, infection or inflammation are other possible reasons [11,16].

### 4.2. Prevalence

According to the existing literature, only a few papers have reported the prevalence of the cyst of the canal of Nuck in children. Paparella et al. described a prevalence of 0.74% in 353 1–14-year-old female patients with inguinal swellings [27]. A similar finding was shown by Akkoyun et al., who reported a prevalence of 0.76% in a cohort of 0–16-year-old girls [23]. Huang et al. found a prevalence of the cyst of the canal of Nuck in 1% of girls aged from 1 month up to 14 years [28]. No data about the prevalence in adults are available. Therefore, no valid statement about the prevalence in adult females can be made as of yet.

### 4.3. Classification

**Figure 5:**
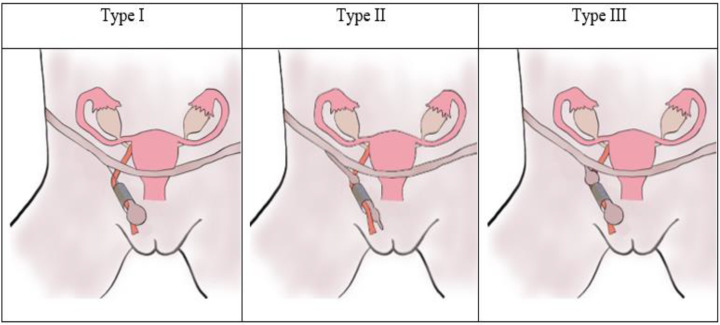
Schematic overview of the different types of cysts of the canal of Nuck as classified by Counseller and Black.

The commonly used classification was developed by Counseller and Black, who classified cysts of the canal of Nuck into three different types [29]. The most prevalent type forms a hydrocele along the round ligament without any communication with the peritoneal cavity. The second type communicates with the peritoneal cavity, and the third, the “hour-glass” type, is constricted by the inguinal ring, whereby one part communicates with the peritoneal cavity, while the other part does not. The three different types of cyst of the canal of Nuck are shown in Figure 5.

A recent classification was published by Wang et al. in 2021 [30], who subdivided the cyst of the canal of Nuck into four groups according to its anatomical position. Type A is located subcutaneously over the inguinal canal, Type B resides inside the inguinal canal, Type C is confined to the internal inguinal ring, and Type D extends from the internal inguinal ring to the inguinal canal or subcutaneously.

### 4.4. Clinical Presentation and Diagnostic Methods

The clinical presentation of a cyst of the canal of Nuck shows an inguinal or genital, painless or painful swelling, with no attending nausea or vomiting [5,6,7,8,9,10,11,12,13,14,15,16,17,18,19,20,21,22]. Some existing reports identify that the mass can be reduced manually [8,17,19], and shows no increase in volume when performing the Valsalva maneuver [14,19]. Several authors report an increase in the volume of the swelling when the patient is standing [13,17], while others do not [6]. In general, a differentiation from other entities that cause inguinal or genital swelling based on symptoms and physiological examination is not possible. The most important differential diagnosis of the cyst of the canal of Nuck is the inguinal hernia, with which it is often initially mistaken [9,12,23]. The co-existence of an inguinal hernia is reported in up to 40% of patients with a cyst of the canal of Nuck, making diagnosis even more difficult [16,17,18]. Hydroceles that extend to the vulva may initially be mistaken for Bartholin cysts. [6,7]. Other differential diagnoses for the cyst of the canal of Nuck are lymphadenopathy, cold abscesses, endometriosis of the round ligaments, ganglion cysts, varicosity of the round ligament and other vascular diseases, or neoplasms such as lipomata or leiomyomata [3,10,17,20,21]. An overview of important differential diagnoses of the cyst of the canal of Nuck is presented in Table 1.

Radiological imaging is of utmost importance in distinguishing from the differential diagnoses presented above. For initial imaging of suspect inguinal or genital swellings, sonography is the preferred investigative method. On ultrasound, the cyst of the canal of Nuck appears as a thin-walled anechoic or hypoechoic formation with no changes on Valsalva maneuver and lack of vascular flow on color Doppler [10,15,16,21]. In addition to high-resolution sonography, cross-sectional imaging should be performed to obtain detailed information about the cyst formation. The method of choice should be MRI, which enables a more precise view of anatomical conditions with no radiation compared with the computed tomography (CT). On MRI, the cyst of the canal of Nuck represents as a thin-walled mass, which appears hypointense on T1-weighted sequences and hyperintense on T2-weighted ones [1,16,21,22]. After contrast administration, no enhancing of the cystic mass, which is considered a sign of benignity, can be observed within MRI [17,31,32]. Due to its radiation, CT is only the second-line method, when accessing inguinal or genital swellings, in particular when imaging children. On CT, the cyst of the canal of Nuck appears as a homogeneous fluid-filled unilocular sac extending along the course of the round ligament [13,15,19]. As with MRI, no enhancement of the interior cyst can be identified within CT-imaging after contrast administration [15,33].

### 4.5. Treatment

Due to the extreme rarity of cysts of Nuck’s canal, no standard therapeutic procedure as yet exists. Although conservative therapy options such as aspiration or sclerotherapy of female hydroceles are reported in the literature, hydrocelectomy is recommended, with or without ligation of the cyst, as treatment of choice [6,7,10,15,19,21,22,56]. Due to anatomical conditions or pathologies such as necrosis of the round ligament, a radical excision of it may be necessary, in addition to hydrocelectomy [9,17,22]. Several authors suggest repairing intraoperative defects by using a polyurethane mesh [5,22,32]. In addition to hydrocelectomy, an aesthetic correction of the vulva may be required as part of the surgery, if the hydrocele has extended to the labia majora [6,7].

Recent therapeutic approaches consider laparoscopic intervention for the treatment of female hydroceles [8,30,57,58]. In particular, the transabdominal preperitoneal (TAPP) and the totally extra-peritoneal (TEP) techniques are considered as popular laparoscopic methods, which can also be used in the treatment of cyst of the canal of Nuck [8,12,30,58]. Laparoscopy can not only be used as a treatment option, but also as an additional diagnostic method to determine the hydrocele and anatomical conditions. Compared with TEP, TAPP has a better diagnostic potential, due to the improved imaging of the abdominal cavity, its anatomical variations and the hydrocele itself [8,12,30,58]. However, sometimes difficult anatomical conditions may prevent a laparoscopic repair, requiring conversion to traditional open anterior surgery [59]. Furthermore, laparoscopic intervention requires a mesh prosthesis to repair the defect within the abdominal wall [8,30,58]. Therefore, the surgical intervention of a cyst of the Nuck’s canal must be well considered and adapted in advance of to the anatomical conditions, as well as the skill of the surgeon.

## 5. Discussion

A hydrocele in young females is a very uncommon disease, and it occurs even more rarely in adult women. Due to its infrequency, many health professionals are not even aware of its existence. Therefore, it is of utmost importance to raise awareness of clinicians, including surgeons and radiologists on the presence of the canal of Nuck as well as the possibility of a female hydrocele.

The primary symptom of the cyst of the canal of Nuck is a painless or painful swelling in the groin or the labia majora [1,2,7,8,10,11,22]. Initially, in many of the reported cases in the literature, they were wrongly suspected of being an inguinal hernia [9,12,23], which is the most common differential diagnosis of the cyst of the canal of Nuck. Through well-targeted physical examination, followed by high-resolution sonography, a differentiation of a cyst of the canal of Nuck from other entities can easily be performed. With MRI, the anatomical condition can be clarified and the diagnosis of cyst of the canal of Nuck determined. Therefore, in our opinion, an MRI investigation for suspect inguinal or genital swelling should be mandatory.

The treatment of choice is surgical excision of the cyst. Based on our literature research, only a few reports about surgical approaches are available. However, due to the rarity of a Nuck’s canal cyst, there is no defined standard method of intervention to date. In our opinion, the surgical approach should be adapted, based on the type of cyst of the canal of Nuck, the anatomical conditions, and the experience of the responsible surgeon. Although laparoscopic approaches have advantages such as reduced blood loss, less wound drainage, and a better aesthetic outcome, they are associated with higher risk for peri- and postoperative complications such as enterotomy, bowel injury, postoperative bleeding and ileus [60]. Thus, it is of utmost importance to consider the risk-benefit ratio before surgery. Due to the variation of the hydrocele with no connection to the peritoneal cavity, it was decided to perform an open ligation and hydrocelectomy in the present case, followed by repair of the inguinal defect without the use of mesh.

In the available literature, only a few reports of cysts of the canal of Nuck have been published since its discovery [5,6,7,8,9,10,11,12,13,14,15,16,17,18,19,20,21,22]. However, in recent years, the number of published cases regarding this topic has increased significantly. One possible reason is the improvement in imaging methods, which enables a better overview of this anomaly and its anatomical features.

In conclusion, the cyst of the canal of Nuck in adult women is an extremely rare disease. If one is aware of its existence, it can be easily diagnosed using modern diagnostic methods and treated by adequate surgical approaches. A cyst of the canal of Nuck should always be considered as a possible cause in suspect inguinal and genital swellings in females.

## 6. Conclusions

Due to the rare clinical occurrence and the lack of literature, a diagnosis of a cyst of the canal of Nuck is often difficult to make, not only for inexperienced surgeons, but also for medical experts. Thus, interdisciplinary collaboration in healthcare between various different fields, such as radiology and surgery, is necessary to prevent misdiagnosis as well as resultant errors in treatment. A focused physical examination followed by high-resolution sonography enables the diagnosis of a cyst of the canal of Nuck. To plan an adequate surgical intervention, cross-sectional imaging, preferably MRI, allowing clarification of the anatomical conditions is of utmost importance. Our review provides insight into the anatomical background, diagnostics, and surgical intervention of a cyst of the canal of Nuck. This article may serve as the foundation for raising awareness about the possibility of female hydroceles and provide guidelines for diagnostic and surgical methods.

## Figures and Tables

**Table 1:** Comparison of the Cyst of the canal of Nuck and possible differential diagnosis.

Differential Diagnosis	Symptoms	Physical Examination	Imaging
Cyst of the canal of Nuck	Cysts of the canal of Nuck present as an inguinal or genital, painless or painful swelling with no gastrointestinal symptoms.	On clinical examination, an inguinal or genital swelling is palpable. This mass shows no increase in volume by performing the Valsalva maneuver, and may is manually reducible.	On ultrasound, an anechoic or hypoechoic lesion without changes in the Valsalva maneuver and without vascular flow can be detected. Computed tomography (CT) imaging shows the cyst of the canal of Nuck as a homogeneous fluid-filled lesion along the round ligament. On magnetic resonance imaging (MRI), it shows as a thin-walled lesion, which appears hypointense on T1-weighted and hyperintense on T2-weighted sequences.
Inguinal hernia [34,35,36,37]	Inguinal hernias may be asymptomatic or symptomatic along with swelling, discomfort, or pain in the groin. Activities that increase intra-abdominal pressure may increase these symptoms. Sudden severe pain, nausea and vomiting indicate possible incarceration of organs in the hernia sac.	The gold standard for hernia diagnostics is clinical examination, although diagnosis is more difficult in women. A female inguinal hernia is confirmed if a bulge is palpable with the open hand over the groin during a Valsalva maneuver.	Although it is rarely necessary, imaging may be useful in unclear situations. Ultrasound is a highly sensitive method of identifying hernias. While the herniating fat appears hyperechoic on ultrasound, the bowel may show peristalsis. Valsalva maneuver is an important examination during the sonography, to increase the swelling and show characteristic movement of the herniating tissues. When ultrasound is not sufficient, a dynamic MRI or CT can be considered. On MRI, hernias present as pathological widening of the anteroposterior inguinal canal and/or protrusion of gastro-enteric content within the inguinal canal.
Lymph-adenopathy [38,39]	Localized inguinal lymphadenopathy is typically caused by infection, while malignancy rarely presents itself solely in the inguinal lymph nodes.	Inguinal lymph node size over 1.5 cm should be suspected as pathological. Pain and tenderness on a lymph node is a non-specific finding. Lymphadenopathies resulting from infections are usually free moving. Acute inflammation makes nodes tauter with concomitant tenderness, while chronic inflammation leads to hard nodes. Painless and adamantine nodes, which are fixed to the surrounding tissues are usually caused by metastatic cancer or granulomatous diseases. Rubbery mobile nodes are typical of lymphoma.	On ultrasound, benign lymphadenopathies emerge as ovoid lesions with various borders, inconspicuous hilum, and isoechoic internal echogenicity, while neoplastic disorders present as round lesions with a sharp border, no hilum and a hypoechoic internal echogenicity. Most benign lymphadenopathies present as ovoid lesions with a central fatty hilum on CT and MRI. Round morphology in addition to changes in size, signal intensity, and dynamic gadolinium contrast enhancement are typical for malignant lesions.
Bartholin cysts [40,41,42]	Bartholin cyst can be asymptomatic or symptomatic, associated with painful swelling in not only the genital but also the inguinal region.	During examination small asymptomatic cysts may be observed as small masses, while larger cysts and abscesses are associated with cellulitis, severe pain and swelling. In addition, a Bartholin gland abscess presents with erythema, edema and sometimes ruptured skin.	On ultrasound, a Bartholin cyst is a central hypoechoic to anechoic lesion, surrounded by a stronger reflective cystic wall, which presents as increased echo enhancement on the posterior side. On MRI, it shows as a small cystic mass with a high signal intensity on T2-weighted sequences and also, depending on the mucoid content, on T1-weighted ones.
Endometriosis of the round ligament [43,44]	If endometriosis affects the extra pelvic portion of the round ligament it may present as a painful, palpable groin mass with or without menstrual variation.	On examination, the endometriosis of the round ligament presents as groin mass without fluctuation, which is non-reducible and possibly partially tender. There are no changes through straining, coughing or adjustments in the patient’s position.	On sonographic examination, endometriosis of the round ligament shows as an inhomogeneous hypoechoic lesion with poorly defined boundaries. On MRI scans, endometriosis of the round ligament, which usually presents as a thin hypointense structure, appears thickened, shortened and irregular. While pure fibrous lesions are hypointense on T1- and T2- weighted sequences, hemorrhagic lesions are hyperintense on T1-weighted images.
Ganglion cysts [45,46]	Ganglion cysts of the hip joint are usually asymptomatic but may cause pain through compression of nerves and vessels. They may present as swelling of the groin or genital region, when they become larger.	On clinical examination, ganglion cysts show as tender, non-pulsatile masses, which may limit the range of motion of the hip joint. Deeper cysts are more difficult to palpate and usually a radiological imaging is necessary to detect them.	Ganglion cysts present on ultrasound as a hypoechoic lesion without the ability to identify the exact joint connection. On CT, ganglion cysts show lower attenuation than muscles, but higher ones than fat. After contrast administration, a rim enhancement may be observed. MRI can show round or ovoid cystic masses with low signal intensities on T1- and high ones on T2-weighted sequences. Similar to CT, a rim enhancement can be observed on T1-weighted MRI sequences, after contrast administration.
Lipomata [47]	Lipoma presents as a painless soft-tissue mass. Deeper ones may be larger and present as asymmetrical.	On palpation, lipomata are freely movable doughy subcutaneous masses. Deeper ones, namely intramuscular lipomata move simultaneously with muscle contraction.	On ultrasonography, lipomata present as homogenous hyperechogenic masses. MRI is necessary for any deeper lipomas or lesion bigger than 5 cm. Lipomata show as homogenous lesions, isointense to fat and may contain thin fibrous septae, on MRI. CT is only the second choice, when patients are inept for MRI due to medical reasons.
Leiomyomata [48,49,50]	Leiomyomata may arise in unusual regions such as the vulva. Clinically, these vulvar leiomyomata usually present as painless swellings of the genital region.	On palpation a vulvar leiomyoma shows as a partially mobile mass, which is non-tender in most cases. In certain superficial lesions, a peduncle is sometimes palpable.	On ultrasound leiomyomata present as well-defined, solid, concentric, hypoechoic masses. Due to its bad soft-tissue contrast, CT has a minor role in diagnostics. Vaginal Leiomyomata are isotense to muscles on T1-weighted MRI sequences and enhance homogenously after contrast administration. On T2-weighted sequences, vulvar leiomyomata show a low signal intensity similar to that of smooth muscles.
Varicosity of the round ligament [51,52,53,54,55]	Varicosity of the round ligament is a further rare condition, which commonly occurs as painful or painless inguinal swelling. In most common cases, the occurrence is associated with pregnancy.	On physical examination, varicosity of the round ligament occurs as a soft groin mass. Some authors describe an increase in size of the mass in standing position and on Valsalva maneuver.	The sonographical characteristics of the round ligament varicosities are dilated veins that appear as multiple echo free serpentine tubes, some of which drain into the inferior epigastric artery, with no herniated bowel or lymphadenopathy. Doppler sonography reveals hypervascularity with a venous flow pattern which increases during Valsalva-maneuver. On both MRI and CT, the varicosity of the round ligament appears as a well-defined serpentine structure extending along the inguinal course of the round ligament of the uterus. In the case of thrombosis, the MRI will show a high T1 and a low T2 signal.

## Data Availability

For data requests, please contact the corresponding author.
